# Correction: Using machine learning to understand age and gender classification based on infant temperament

**DOI:** 10.1371/journal.pone.0316132

**Published:** 2024-12-17

**Authors:** Maria A. Gartstein, D. Erich Seamon, Jennifer A. Mattera, Michelle Bosquet Enlow, Rosalind J. Wright, Koraly Perez-Edgar, Kristin A. Buss, Vanessa LoBue, Martha Ann Bell, Sherryl H. Goodman, Susan Spieker, David J. Bridgett, Amy L. Salisbury, Megan R. Gunnar, Shanna B. Mliner, Maria Muzik, Cynthia A. Stifter, Elizabeth M. Planalp, Samuel A. Mehr, Elizabeth S. Spelke, Angela F. Lukowski, Ashley M. Groh, Diane M. Lickenbrock, Rebecca Santelli, Tina Du Rocher Schudlich, Stephanie Anzman-Frasca, Catherine Thrasher, Anjolii Diaz, Carolyn Dayton, Kameron J. Moding, Evan M. Jordan

The second author’s name is spelled incorrectly. The correct name is: Erich Seamon. The correct citation is: Gartstein MA, Seamon E, Mattera JA, Bosquet Enlow M, Wright RJ, Perez-Edgar K, et al. (2022) Using machine learning to understand age and gender classification based on infant temperament. PLOS ONE 17(4): e0266026. https://doi.org/10.1371/journal.pone.0266026

There is an error in affiliation 2 for author Erich Seamon. The correct affiliation 2 is: Institute for Modeling, Collaboration, and Innovation, University of Idaho, Moscow, Idaho, USA.
