# Supplementary figures and images for: Using machine learning to understand age and gender classification based on infant temperament
Source: PLoS One. 2022 Apr 13;17(4):e0266026. doi: 10.1371/journal.pone.0266026 (PMC9007342; doi:10.1371/journal.pone.0266026)

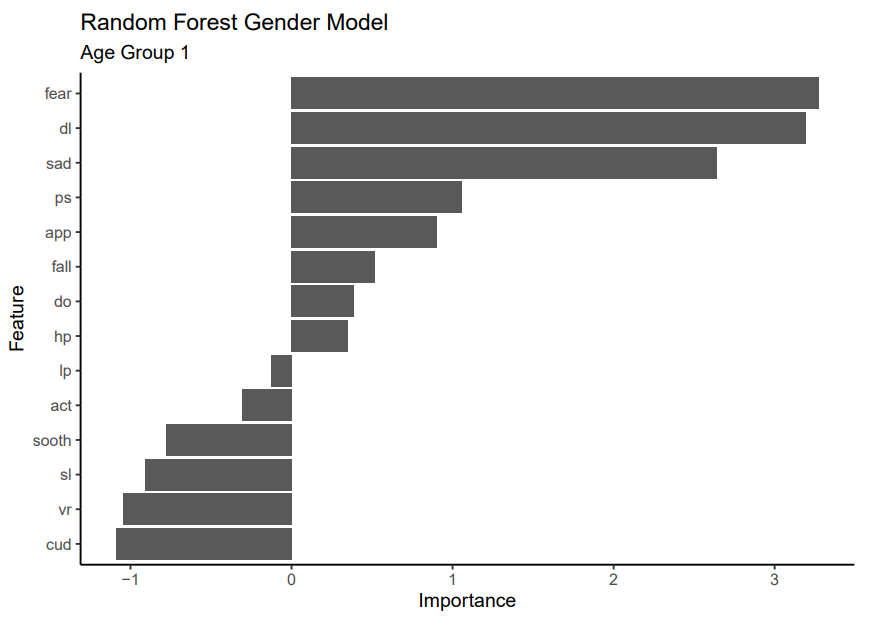

Supplement: S1 Fig — DL–distress to limitations; Sad–sadness; PS–perceptual sensitivity; App–approach; Fall–falling reactivity; DO–duration of orienting; HP–high intensity pleasure; LP–low intensity pleasure; Act–activity level; Sooth–soothability; SL–smiling and laughter; VR–vocal reactivity; Cud–cuddliness. (TIF) [file pone.0266026.s001.tif]

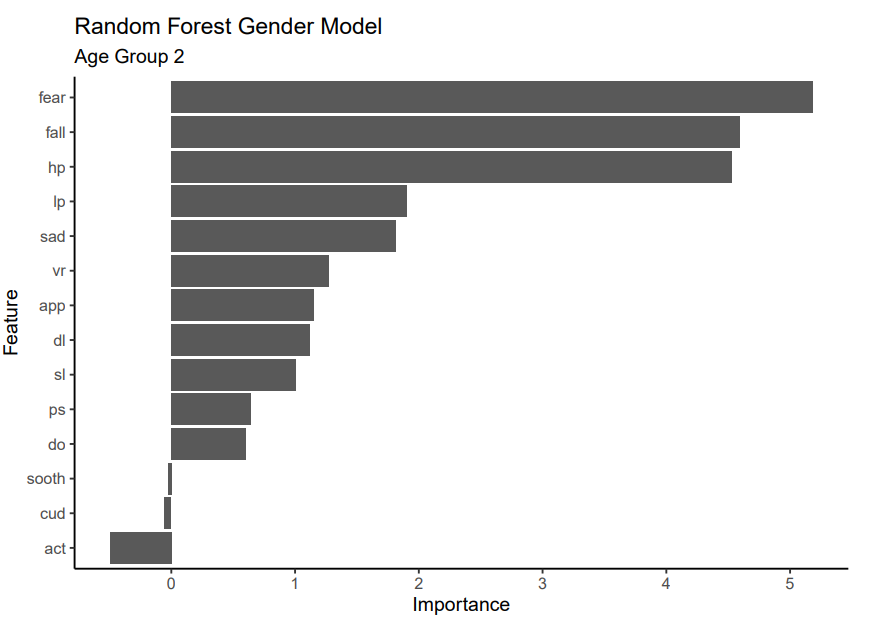

Supplement: S2 Fig — Fall–falling reactivity; HP–high intensity pleasure; LP–low intensity pleasure; Sad–sadness; VR–vocal reactivity; App–approach; DL–distress to limitations; SL–smiling and laughter; PS–perceptual sensitivity; DO–duration of orienting; Sooth–soothability; Cud–cuddliness; Act–activity level. (TIF) [file pone.0266026.s002.tif]

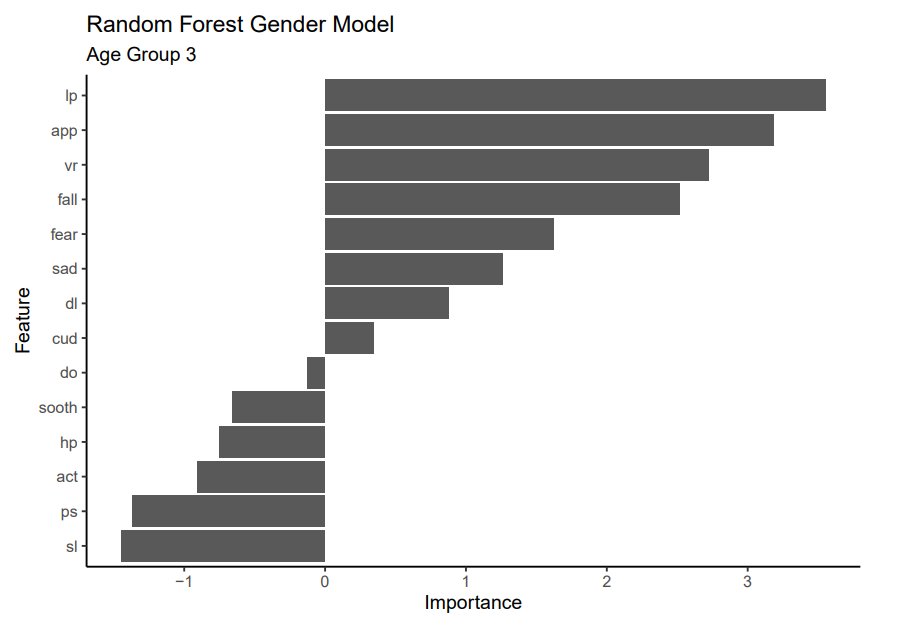

Supplement: S3 Fig — LP–low intensity pleasure; App–approach; VR–vocal reactivity; Fall–falling reactivity; Sad–sadness; DL–distress to limitations; Cud–cuddliness; DO–duration of orienting; Sooth–soothability; HP–high intensity pleasure; Act–activity level; PS–perceptual sensitivity; SL–smiling and laughter. (TIF) [file pone.0266026.s003.tif]
